# Nanomaterial payload delivery to central nervous system glia for neural protection and repair

**DOI:** 10.3389/fncel.2023.1266019

**Published:** 2023-10-24

**Authors:** Jayant Saksena, Adelle E. Hamilton, Ryan J. Gilbert, Jonathan M. Zuidema

**Affiliations:** ^1^Department of Biomedical Engineering, Rensselaer Polytechnic Institute, Troy, NY, United States; ^2^Center for Biotechnology and Interdisciplinary Studies, Rensselaer Polytechnic Institute, Troy, NY, United States; ^3^Department of Materials Science and Engineering, Rensselaer Polytechnic Institute, Troy, NY, United States; ^4^Albany Stratton Veterans Affairs Medical Center, Albany, NY, United States; ^5^Department of Biochemistry and Molecular Pharmacology, Mario Negri Institute for Pharmacological Research IRCCS, Milan, Italy

**Keywords:** nanomaterial, nanoparticle, astrocyte, oligodendrocyte, microglia, drug delivery, central nervous system, glia

## Abstract

Central nervous system (CNS) glia, including astrocytes, microglia, and oligodendrocytes, play prominent roles in traumatic injury and degenerative disorders. Due to their importance, active pharmaceutical ingredients (APIs) are being developed to modulate CNS glia in order to improve outcomes in traumatic injury and disease. While many of these APIs show promise *in vitro,* the majority of APIs that are systemically delivered show little penetration through the blood–brain barrier (BBB) or blood-spinal cord barrier (BSCB) and into the CNS, rendering them ineffective. Novel nanomaterials are being developed to deliver APIs into the CNS to modulate glial responses and improve outcomes in injury and disease. Nanomaterials are attractive options as therapies for central nervous system protection and repair in degenerative disorders and traumatic injury due to their intrinsic capabilities in API delivery. Nanomaterials can improve API accumulation in the CNS by increasing permeation through the BBB of systemically delivered APIs, extending the timeline of API release, and interacting biophysically with CNS cell populations due to their mechanical properties and nanoscale architectures. In this review, we present the recent advances in the fields of both locally implanted nanomaterials and systemically administered nanoparticles developed for the delivery of APIs to the CNS that modulate glial activity as a strategy to improve outcomes in traumatic injury and disease. We identify current research gaps and discuss potential developments in the field that will continue to translate the use of glia-targeting nanomaterials to the clinic.

## 1. Introduction

Glia, which constitute roughly half the cells in the central nervous system (CNS), have essential yet distinct roles in supporting neuronal homeostasis and signal transduction (Allen and Lyons, 2018). In particular, astrocytes, microglia, and oligodendrocytes are necessary for regulating synaptic function, contributing to metabolic support, creating myelin sheaths for signal transduction, and in the CNS immune response (Somjen, 1988; Tomassy et al., 2016; von Bartheld et al., 2016). Glia also are critical players in disease and after traumatic injury, as microglia are the primary source of pro-inflammatory cytokines, astrocytes are regulators of synaptic homeostasis and glial scar formation after injury, and demyelination or changes in myelin thickness by oligodendrocytes alters signal conduction speed (Colonna and Butovsky, 2017; Liddelow and Barres, 2017; Wang S. S. et al., 2018). Due to the fact that neuronal regeneration is highly restricted following CNS injury and disease, glia have begun to emerge as important targets in the development of active pharmaceutical ingredients (APIs) in order to improve clinical outcomes. However, since many APIs do not readily cross the blood–brain barrier (BBB) to impart their action on glia, nanomaterials have been engineered to carry APIs to the site of action, extend the API release timeframe, and also to impact cellular behavior based on their architectural features (Zhang et al., 2016; Dai et al., 2021).

Nanomaterials, including nanoparticles and nanostructured scaffolds, offer many advantages in the delivery of APIs to CNS glia. Nanoparticles (NPs), composed of polymers, liposomes, inorganic materials, and extracellular vesicles, are used to deliver APIs to glia because they can be administered systemically, engineered to cross the BBB, carry and protect sensitive APIs, and be targeted to cells or regions of interest using antibodies, targeting peptides, and even nucleic acids (Patel et al., 2012; Mann et al., 2016; Zuidema et al., 2016; Furtado et al., 2018; Zhou et al., 2018; Ciciriello et al., 2022; Waggoner et al., 2022). The advantage of NP technologies is that they can be administered systemically; however, in some cases, such as TBI, BBB permeability can decrease over time, which can reduce the accumulation of the API in the CNS at longer time points after injury (Werner and Engelhard, 2007; Mann et al., 2016). In CNS disorders, BBB breakdown often occurs prior to neurodegeneration and persists as the disease progresses (Sweeney et al., 2018a,b). While this can be advantageous for API delivery to the CNS, there are also complex mechanisms, including disrupted BBB transporter expression, inflammation, immune products, and impaired solute transport, which can limit API accumulation in these regions (Sweeney et al., 2018b). NP design needs to consider this when attempting to traverse the injured or diseased BBB to deliver APIs to regions of interest. Nanostructured scaffolds have other advantages, even though they generally must be surgically implanted or injected into the site of interest. The nanoscale topographical features of nanomaterial scaffolds can influence cellular function, migration, and growth; APIs can be delivered from either the surface of the scaffold or incorporated into the scaffold to extend release, and, since the scaffolds are implanted directly at the site, APIs are released locally to glia (Tsui et al., 2019; Puhl et al., 2020, 2022). Here, we present the current state-of-the-art in API delivery to CNS glia using nanomaterials, point out the existing gaps in the research, and discuss the potential future developments and advances of this field that will drive nanomaterial delivery of APIs to CNS glia towards the clinic.

## 2. Nanomaterials for API delivery to CNS glia

### 2.1. Nanoparticle API delivery to astrocytes

Astrocytes are CNS glia that perform core homeostatic functions and whose radiating processes can contact upwards of 1 million synapses in humans (Hasel and Liddelow, 2021). They are integral parts of the BBB where they uptake metabolites such as glucose to fuel active neurons, modulate neurotransmitter concentration in synapses, phagocytose synapses, form part of the glymphatic system, and aid in the homeostatic control of neuronal redox stress (Sofroniew and Vinters, 2010; Hasel and Liddelow, 2021). In the event of injury or pathology, including stab wound injuries, experimental autoimmune encephalomyelitis (EAE), middle cerebral occlusion (MCAO), hypertrophic ciliary neurotrophic factor induction, cortical lesion, spinal cord injury (SCI), Alzheimer’s disease, Parkinson’s disease, prion disease, Huntington’s disease, multiple sclerosis (MS), and amyotrophic lateral sclerosis (ALS), astrocytes respond via a process termed reactive astrogliosis (Anderson et al., 2014; Pekny et al., 2016; Escartin et al., 2019). This process can be protective, but persistent reactive astrogliosis can become maladaptive, making it a target for APIs (Pekny et al., 2016). Therefore, APIs that act on astrocytes have been developed that target metabolic pathways, transporters and receptors, cell–cell interactions, and even as glia-to-neuron conversion therapies (Lee et al., 2022). Their role in disease and trauma, as well as recent advances in nanomaterial API delivery, make astrocytes a critical cell to target in order to improve clinical outcomes in CNS disease and injury.

Nanoparticles (NPs), including polymer, dendrimer, lipid, and inorganic nanoparticles, have all been developed to deliver APIs to astrocytes. Specific nanoparticle types, their payloads, the model studied, and the outcomes are listed in Table 1. The majority of astrocyte research to date has employed either polymer NPs or dendrimers to deliver APIs (Newland et al., 2014; Serramia et al., 2015; Lozic et al., 2016; Kong et al., 2017; Chowdhury et al., 2018; Surnar et al., 2018; Holmkvist et al., 2020; Proulx et al., 2020; Vismara et al., 2020; Wang et al., 2020; Clementino A. et al., 2021; Gu et al., 2022; Huang et al., 2022; Narsineni et al., 2023; Perumal et al., 2023; Sabourian et al., 2023; Zhang F. et al., 2023). API payloads range from small molecule drugs to proteins, plasmid DNA, and siRNA (Montenegro et al., 2011; Kannan et al., 2012; Chen and Foldvari, 2016; Tickle and Chari, 2019; Porkolab et al., 2020; Gu et al., 2022). The goal of most APIs is to push astrocytes towards a more protective phenotype, improving their ability to protect neurons in these environments. This includes reducing reactive oxygen species, decreasing astrocyte inflammatory response, and reducing inflammatory cytokine release (Table 1). NP API delivery to astrocytes has been shown to improve outcomes in cerebral palsy, blast-induced hearing loss, neural implants, ALS, and SCI models (Table 1), demonstrating the potential of these therapeutic strategies in future clinical applications.

**Table 1:** Studies that use nanoparticle delivered APIs to target central nervous system glia.

Material	Payload	Study of Bioactivity	Outcome	References
Astrocytes				
Arginine-modified PEI and Poly(lactide-*co*-glycolide)	Plasmid DNA	*In vitro* human astrocytes	Arginine PEI increased plasmid DNA transfection in astrocytes	Proulx et al. (2020)
Cell adhesion peptide-conjugated gemini nanoplexes	Plasmid DNA	*In vitro* A7 astrocytes, *in vivo* intravitreal injection	Gemini nanoplexes enhanced transfection of astrocytes	Narsineni et al. (2023)
Alanine and glutathione targeted niosomes	Evans blue BSA	*In vitro* primary rat astrocyte cultures	Alanine and glutathione niosomes increased astrocyte uptake in *in vitro* BBB model	Porkolab et al. (2020)
PAMAM dendrimers	2-(3-Mercaptopropyl) pentanedioc acid	*In vivo* cerebral palsy model	Dendrimers localize in activated astrocytes and microglia and improve motor function	Zhang F. et al. (2023)
Transferrin tagged-PEG	Minocycline	*In vivo* blast-induced hearing loss model	Reduction in astrocyte activation	Perumal et al. (2023)
Aquaporin 4 Ab targeted poly(glycidyl methacrylate)	Resveratrol	*In vivo* partial optic nerve transection injury	Reduce oxidative damage and AQP 4 immunoreactivity, preserve visual function	Lozic et al. (2016)
K2®Transfection NPs	BDNF-plasmid	*In vitro* co-culture	A7 astrocytes increased BDNF expression protecting SH-SY5Y cultures	Chen and Foldvari (2016)
Solid lipid nanoparticle	Idebenone	*In vitro* primary rat cerebral cortex astrocytes	Inhibition of ROS production and increase in viability	Montenegro et al. (2011)
Cationic carbosilane dendrimers	HIV-1 NEF siRNA	*In vivo* uptake *via* retro-orbital venous plexus administration	Delivered siRNA to HIV-infected astrocytes	Serramia et al. (2015)
NeuroMag magnetic nanoparticles complexed with plasmids	Reporter protein plasmids	*In vitro* primary rodent astrocyte transfection assay	Levels of transfection using magnetic-multifection reach viral methods	Tickle and Chari (2019)
Poly(lactide-*co*-glycolide)	Minocycline	*In vivo* neural implant model with PLGA NPs incorporated onto gelatin coatings	Delayed and significant reduction in astrocytic response	Holmkvist et al. (2020)
Poly(lactide-*co*-glycolide)-block (*b*) polyethyleneglycol functionalized with terminal lipophilic triphenylphosphonium cation	Aspirin and coenzyme Q10	*In vivo* therapeutic efficacy in SOD1 mice	Increased ATP production and reduced ROS production in astrocytes and neurons	Surnar et al. (2018)
Gold and PAMAM Dendrimer NPs	Gastrodin	*In vitro* astrocyte gene expression	Reduction in astrocyte cytokine release	Huang et al. (2022)
Lecithin/chitosan	NPs only	*In vitro* human astrocytes	Astrocyte viability in psychosine cultures increased	Clementino A. et al. (2021)
Poly(lactide-*co*-glycolide)	Polo-like kinase 4 siRNA	*In vivo* contusion SCI rat model	Locomotor score increased	Gu et al. (2022)
Peptide conjugated chitosan	Plasmid DNA	*In vitro* EAE astrocytes	Large pspCS particles were uptaken preferentially by EAE astrocytes	Kong et al. (2017)
Lipopolysaccharide-bonded chitosan-quantum dots/poly acrylic acid	NP only	*In vivo* mild stab SCI injury	NPs were preferentially uptaken by astrocytes and neurons *in vivo*	Sabourian et al. (2023)
Carboxy-methylchitosan/poly(amidoamine) dendrimer nanoparticle	Methylprednisolone	*In vitro* rat cortical astrocyte cultures	NPs are taken up by astrocyte endocytosis, followed by an increase in frequency of transient exocytotic fusion events	Chowdhury et al. (2018)
Knotted 2-(dimethylamino)ethyl methacrylate and poly(ethylene glycol) methyl ethyl methacrylate polymer	Plasmid DNA	*In vitro* Neu 7 astrocyte cell line	Improved transfection over commercially available controls	Newland et al. (2014)
Valproic acid-labeled chitosan	NP only	*In vivo* contusion SCI injury	Decreased lesion volume, suppression of reactive astrocytes and inflammation	Wang et al. (2020)
Poly(lactide-*co*-glycolide)-*b-*poly(ethylene glycol)-triphenylphosphonium	Antiretrovirals, coenzyme Q_10_, and an asprin pro-drug	*In vivo* EcoHIV and methampetamine-exposed animal model	Astrocyte ROS levels reduced	Surnar et al. (2021)
Polyethylene glycol and polyethylene-amine nanogels	Rolipram	*In vitro* astrocyte CM neurons and *in vivo* compression SCI	Reversed toxic effects on motor neurons *in vitro* and improved early functional recovery after SCI	Vismara et al. (2020)
Microglia				
D-T7-TfR and MG1 peptide targeted polycaprolactone-poly(ethylene glycol) NP	Aspirin	*In vivo* ASD mouse model	NPs targeted microglia, inhibited their activation, and improved behavior	He et al. (2022)
Hydroxyl poly(amidoamine) generation-6 dendrimers	Minocycline	*In vivo* cerebral palsy model	NPs targeted microglia *in vivo* following IV administration	Sharma et al. (2017)
Poly(lactide-*co*-glycolide)	Duloxetine	*In vivo* spinal nerve ligation model	NPs localized to spinal microglia, suppressed their activation, and alleviated mechanical allodynia	Kim et al. (2021)
Exosomes from M2 type primary peritoneal macrophage	Berberine	*In vivo* contusion SCI mouse model	Microglia were induced towards M2 phenotype, motor function was improved	Gao et al. (2021)
Reactive oxygen species-responsive dendrimer-peptide conjugate	p-NRF2 peptide	*In vivo* APP/PS1 mouse model	Reduced ROS levels, alleviated microglia activation, and enhanced cognitive function	Liu et al. (2021)
Microglial BV2 cell membrane shell-human serum albumin core NPs	Flavin mononucleotide	*In vivo* FxFAD mouse model	NPs target microglial, improve inflammatory response, and ameliorated cognitive impairment	Zhang M. et al. (2023)
CDX-Chitosan	Fingolimod	*In vivo* experimental autoimmune encephalomyelitis mouse model	Microglia uptake NPs and regulate the inflammatory response	Sepasi et al. (2023)
Hydroxyl terminated generation-4 PAMAM dendrimer	Sinomenine	*In vivo* rabbit model of pediatric TBI	NPs target microglia and attenuate inflammation	Sharma et al. (2020)
Hydroxyl terminated PAMAM dendrimer	2-(phosphonomethyl)-pentanedioc acid	*In vivo* mouse model of experimental autoimmune encephalomyelitis	NPs preferentially uptaken by microglia causing robust anti-inflammatory activity, improved cognition	Hollinger et al. (2022)
Hydroxyl-terminated PAMAM dendrimer	Fluocinolone acetonide	*In vivo* Royal College of Surgeons rat retinal degeneration model	NPs arrest retinal degeneration and attenuate activated microglia	Iezzi et al. (2012)
Hydroxyl terminated generation-4 PAMAM dendrimer	*N-*(5–2-[2-(5-amino-[1,3,4]thiadiazol-2-yl)-ethylsulfanyl]-ethyl-[1,3,4]thiadiazol-2-yl)-2-phenyl-acetamide	*In vivo* mouse model of Rett syndrome	Reduced glutaminase expression in microglia and selective improvement in mobility	Khoury et al. (2020)
Hydroxyl terminated generation-4 PAMAM dendrimer	*N-*acetyl systeine	*In vivo Mecp2*-null Rett syndrome mouse	Localized to microglia and improved behavioral outcomes	Nance et al. (2017)
Hydroxyl terminated generation-4 PAMAM dendrimer	Triamcinolone acetonide	*In vivo* mouse model of oxygen induced retinopathy	Suppresses activated microglia and improves visual function	Cho et al. (2021)
Docosahexaenoic acid nanostructured lipid carrier modified with chitosan and TAT	Glial cell-derived neurotrophic factor, vascular endothelial growth factor	*In vitro* HMC3 microglia cell line	Counteracted inflammatory response in LPS stimulated cultures	Hernando et al. (2022)
Poly(ethylene glycol)-poly-ε-caprolactone	Minocycline	*In vivo* contusion spinal cord injury mouse model	Acutely reduces pro-inflammatory response in microglia and improved behavioral outcomes	Papa et al. (2016)
Amphiphilic poly(amidoamine)dendrimer	siRNA	*In vitro* primary rat microglia cultures	Effectively delivered siRNA and decreased target gene and protein expression	Ellert-Miklaszewska et al. (2019)
Plant-derived extracellular vesicles	Dexamethasone	*In vitro* BV-2 microglial cultures	Inhibited NO production	Ishida et al. (2023)
Lecithin/chitosan nanoparticles	Simvastatin	*In vitro* human macrophage THP-1 cells activated with LPS	Suppression of pro-inflammatory signaling	Clementino A. R. et al. (2021)
Poly(lactide-*co*-glycolide)	Inhibitor of kappa B nuclear factor-kappa B kinase subunit beta siRNA	*In vivo* spinal nerve ligation rats	Mechanical allodynia and secretion of pro-inflammatory mediators reduced	Lee et al. (2021)
Liposomes	Interleukin-4	*In vivo* controlled cortical impact mouse model	Boosted a beneficial microglia phenotype and protected against neuronal loss	Pu et al. (2023)
Astrocyte extracellular vesicles	lincRNA-Cox2 siRNA	*In vivo* LPS-induced mouse microglial proliferation model	Decreased LPS-induced microglial proliferation	Liao et al. (2020)
Mannose functionalized curdlan-based NP	NF-κB p65 siRNA	*In vivo* mouse model of transient middle cerebral artery occlusion	Microglia transitioned to M2 phenotype, reduced neurological deficit score, and increased density of neurons	Ganbold et al. (2020)
Liposome	Macrophage migration inhibitory factor	*In vivo* contusion SCI rat model	Preservation of white matter integrity	Saxena et al. (2015)
Poly(lactic-*co*-glycolic acid)	miRNA-129-5p	*In vitro* BV-2 microglia	Polarized activated microglia into more pro-regenerative phenotype	Kalashnikova et al. (2023)
Lipid nanoparticle	Toll-like receptor 4 siRNA	*In vivo* mouse model of transient middle cerebral artery occlusion	Significant knockdown of TLR4 expression and improved neurological function	Ganbold et al. (2022)
Poly(lactic-*co*-glycolic acid)	Perampanel	*In vivo* photothrombic stroke rat model	Increased M2 polarization, decreased size of infarct, and increased motor function	Shin et al. (2022)
Polyamidoamine dendrimer	Triamcinolone acetonide	*In vivo* peripheral nerve injury model	Targeted microglia and reduced mechanical allodynia	Kim et al. (2017)
Poly (ethylene glycol)-*block*-poly (D,L-lactide)	C3-siRNA	*In vivo* middle cerebral artery occlusion mouse model	Decreased C3 expression in microglia and reduced volume of ischemic zone	Wang Y. et al. (2018)
Mannose functionalized DoGo Lipid nanoparticle	Toll-like receptor 4 siRNA	*In vitro* BV2 microglia	Silencing of TLR4 and polarization towards M2 microglia	Xiao et al. (2021)
Poly(ethylene glycol)-poly-ε-caprolactone	Minocycline	*In vivo* contusion SCI mouse model	Reduction of the pro-inflammatory milieu	Papa et al. (2013)
Poly(ethylene glycol)-Poly(lactic-*co*-glycolic acid) coated with a lipid film	Toll-like receptor 4 siRNA	*In vivo* LPS-injection model	Reduction in microglial activation after LPS injection	Guo et al. (2022)
Poly(ethylene glycol)-poly caprolactone miktoarm star-derived polymersomes	Fisetin	*In vitro* HMC3 human microglia	Reduced ROS and ERK1/2 phosphorylation	Baghbanbashi et al. (2022)
Hydroxyl terminated generation-4 PAMAM dendrimer	N-acetyl cysteine	*In vivo* mouse model of ischemia-induced neonatal white matter injury	Reduces the detrimental pro-inflammatory response	Nance et al. (2015)
Poly(lactic-*co*-glycolic acid) and _L_-tyrosine polyphosphate	Rolipram	*In vitro* primary microglia	NPs did not induce release of proinflammatory cytokines	Cahalane et al. (2020)
Fas ligand antibody conjugated PEGylated-lipid nanoparticle	3-n-Butylphthalide	*In vivo* mouse middle cerebral artery occlusion model	Accumulation in microglia and improvement in neurological deficit	Lu et al. (2014)
Oligodendrocytes				
Transferrin receptor binding peptide conjugated lipid nanocapsule with super paramagnetic iron oxide nanoparticle	Retinoic acid	*In vitro* oligodendrocyte progenitor cell culture	Induced differentiation to more mature, myelin produced oligodendrocytes	Moura et al. (2023)
Liposomes	Interleukin-4	*In vitro* OPC culture and *in vivo* TBI mouse model	IL-4 induced mature, myelin producing oligodendrocytes and improved sensorimotor neurological recovery following TBI	Pu et al. (2021)
HEK293T extracellular vesicles	miR-219a-5p	*In vitro* OPC culture and *in vivo* experimental autoimmune encephalomyelitis mouse model	Induced OPC differentiation and improved EAE functional outcomes	Osorio-Querejeta et al. (2020)
NG-2 Ab conjugated Poly(lactic-*co*-glycolic acid)	Leukaemia inhibitory factor	*In vitro* OPC culture and *in vivo* mouse model of focal CNS demyelination	Induced OPC differentiation into mature oligodendrocytes and increased myelin repair *in vivo*	Rittchen et al. (2015)
Hexagonal bi-pyramid shaped gold nanoparticle	Nanoparticle nanocatalysis	*In vivo* cuprizone mouse model of demyelination	Induced remyelination	Robinson et al. (2020)
Nogo receptor agonist peptide Nep_1-40_ conjugated human serum albumin poly(ethylene glycol)	Methylprednisolone	*In vivo* rat contusion SCI model	Improved behavioral outcomes	Lin et al. (2019)
NFL-TBS.40–63 peptide vectorized lipid nanoparticle	Neurotrophin-3	*In vitro* oligodendrocyte cultures	Potentiated proremyelinating effects	Fressinaud et al. (2020)
NIDPNAV peptide conjugated gold nanoparticles	NIDPNAV peptide	*In vivo* focal demyelination mouse model	Significantly enhanced myelin content	Farhangi et al. (2023)
Lipoidal nanoparticle	Dimethyl fumarate	*In vivo* cuprizone-induced demyelination rodent model	Rejuvenation of the myelin sheaths and improved functional outcomes	Kumar et al. (2018)
Liposomes	Interleukin-4	*In vivo* murine model of transient cerebral ischemia	Improved white matter integrity and functional outcomes	Zhang et al. (2019)

### 2.2. Nanomaterial API delivery to astrocytes

Nanomaterial strategies targeting astrocytes, including electrospun fibers, composite hydrogels, and hybrid materials, address two occurrences in the astrocytic response to injury: (1) to reduce glial scar formation, or (2) to mitigate an established glial scar (Jarrin et al., 2021). Often, these materials contain anti-inflammatory API payloads or exogenous stem cells which assist in promoting a neuroprotective phenotype, by diminishing the inhibitory chemical barrier and promoting restoration of the blood-spinal cord barrier. Polymer nanofibers are fabricated by electrospinning, a process that allows for the creation of biodegradable and biocompatible scaffolds that can be used in neural tissue engineering (Schaub et al., 2016; Cheng et al., 2021). Due to their high surface area and structure, electrospun nanofibers mimic the native extracellular matrix of neural tissue and are hence suited to promote neural regeneration (Tian et al., 2015). Nanofibers are attractive as drug depots for astrocytes because their intrinsic material properties can alter astrocyte activation or direct astrocyte growth (Zuidema et al., 2014, 2018). Improved astrocyte activation outcomes have been demonstrated by employing nanofiber scaffolds alone, incorporating stem cells with nanofibers, and even with conductive nanofibers (Zhao et al., 2018; Shu et al., 2019; Yan et al., 2020; Dai et al., 2023; Xu et al., 2023). As a means to further promote neural repair and mitigation of secondary injury, nanofibers can be loaded with APIs that act on astrocytes due to their porous nature (Zhang et al., 2021). Growth factors and small molecule drugs released from nanofibers have shown the ability to reduce astrocyte activation (Zhang et al., 2018; Bighinati et al., 2020; Sun et al., 2020), decreasing GFAP expression and improving outcomes (Table 2).

**Table 2:** Studies that use nanomaterials and nanomaterial delivered APIs to target central nervous system glia.

Material	Payload	Study of bioactivity	Outcome	References
Astrocytes				
PHBV, PLA and Collagen electrospun nanofibers		*In vivo* implanted in adult female Sprague dawley rats with complete T10 hemisectioned spinal cord injury	Decreased expression of GFAP by astrocytes	Zhao et al. (2018)
Gelatin-coated nanofibers, cross-linked by genipin with NT-3 (MNS-G/NT3)	NT-3	*In vitro* rodent neural stem cells *In vivo* implantation in rat with T9 segment spinal cord injury	Inhibition of GFAP+ astrocyte differentiation Negligible GFAP+ astrocytes observed; no glial scar	Sun et al. (2020)
PLLA nanofibers loaded with Ibuprofen and Triiodothyronine (PLLA-Ibu-T3)	Ibuprofen, T3	*In vivo* implanted in female CD/Sprague Dawley rats with T9 contusive spinal cord injury	Reduced astrocyte reaction in ventral horn	Bighinati et al. (2020)
PCL/PSA nanofiber scaffold encapsulating MP	MP	*In vivo* implanted in rats with spinal cord transection at T10	Decreased GFAP expression; increased NF200 and GAP43 expression in astrocytes	Zhang et al. (2018)
PLL_PCL triol-co-sebacic acid-co-BES sodium salt (PPSB) nanofibers with human NSCs	BES	*In vivo* implanted in rats with complete spinal cord transection	Fewer GFAP+ astrocytes	Dai et al. (2023)
Hyaluronic acid, with BDNF loaded, micro-sol particle encapsulated into PLLA (core-shell nanofibers); Type I collagen solution, loaded with BMSCs, onto surface of nanofibers	BDNF	*In vitro* astrocytes *In vivo* implanted at site of spinal cord injury in rat	IL-1β and TNF-ɑ expression in astrocytes downregulated Fewer GFAP+ astrocytes	Xu et al. (2023)
PPy embedded into PLA nanoscaffold (PLA/PPy)	PPy	*In vivo* implanted in rat spinal cord injury lesion	Decreased accumulation of GFAP+ astrocyte around injured area	Shu et al. (2019)
Poly [aniline tetramer methacrylamide]-co-[dopamine methacrylamide]-co-[poly(ethylene glycol) methyl ether methacrylate]/PCL (PCAT) with NGF nanofiber mesh	NGF	*In vitro* rodent neural stem cell	Nanofiber mesh applied with electrical stimulation suppressed spreading of differentiated astrocytes	Yan et al. (2020)
Agarose/Gelatin/polypyrrole (Aga/Gel/PPy) (AGP3) – Aga/Gel Hydrogel with PPy nanoparticles	PPy nanoparticles	*In vitro* Primary rodent astrocytes *In vivo* implanted in rats with hemisectioned spinal cord injury	Lower expression of CS56 in astrocytes Decrease in GFAP+ astrocytes in lesion	Yang et al. (2022)
PVA hydrogels with molybdenum sulfide (MoS_2_)/ graphene oxide (GO) nanosheets		*In vitro* neural stem cells *In vivo* implanted in male mice with T9/T10 spinal cord injury	Inhibition of differentiation towards GFAP expressing astrocytes Reduced GFAP expression in lesion	Chen et al. (2022)
Silk-elastin-like-polymer (SELP) (EIS)2-RGD6 (When injected, rapidly forms nanofibrillar hydrogel)	(EIS)2-RGD6	*In vivo* implanted in adult female wistar rats with T10 contusive spinal cord injury	Reduced astrocyte-mediated fibrosis	Gonzalez et al. (2022)
Aligned Silk Fibroin Nanofiber (ASFN) hydrogels + NGF	NGF	*In vivo* implanted in rats with hemisectioned spinal cord injury	Orientational astrocytes along spinal cord	Gao et al. (2022)
PCL nanofibers bound to thiolated hyaluronic acid (HA-SH) and PEGDA in a nanofibrillar hydrogel composite		*In vivo* implanted in adult female Sprague Dawley rats with T9 contusive spinal cord injury	Reduced astrocyte infiltration	Haggerty et al. (2022)
PSS cross-linked CNT and SA (CNT-PSS-SA) with Diacerein (CNT-PSS-SA-DA)	Diacerein	*In vitro* human astrocytes	Reduced expression of IL-6 and IL-1β	Xing et al. (2023)
Chondroitinase ABC (ChABC)-loaded injectable SAP nanostructured hydrogels (Self-organize into braided nanofibers)	ChABC	*In vivo* implanted in rats with T10 weight drop spinal cord injury	Reduced GFAP+ astrocytes in the center of the lesion	Raspa et al. (2021)
Gelatin:Hyaluronic Acid:poly(3,4-ethylenedioxythiophene) polystyrene sulfonate (PEDOT:PSS) (Gel:HA:PEDOT-NPs) nanoparticle hydrogel composite	PSS	*In vivo* implanted in make fisher 344 rats with T3 transected spinal cord injury	Downregulation of GFAP in astrocytes around scaffold activation area	Serafin et al. (2022)
Microglia				
Biodegradable hybrid inorganic nanoscaffolds composed of manganese oxide and coated with laminin	Laminin coating	*In vivo* spinal cord injury site of adult mice	Modulated microglia to reduce scar formation during stem cell transplantation therapy	Yang et al. (2018)
PCL nanofiber scaffolds coated with self-assembled colloidal graphene	Colloidal graphene coating	*In vivo* implanted into the striatum or subventricular zone of adult rats	Reduced microglial infiltration	Zhou et al. (2016)
P(TMC-CL) nanofibrous scaffold		*In vitro* primary microglia from Wistar rat pups	Reduction in microglial phagocytic capacity	Pires et al. (2015)
Self-assembling (RADA)_4_ -IKVAV peptide nanoscaffolds	IKVAV	*In vitro* primary rat microglia *In vivo* intracerebral implantation into Long-Evans rat pups	Microglia remained viable, phagocytosed matrix, ramified with high TNF-ɑ and IL-1b and NO expression and high proliferation Did not lead to microglial migration, proliferation or microglia-induced scarring	Koss et al. (2016)
GNDF-loaded PDA nanoparticle-based anisotropic gelatin scaffolds	GNDF-loaded PDA nanoparticles	*In vitro* murine BV2 microglial cell line	Promote anti-inflammatory M2 microglial phenotype	Ma et al. (2023)
2D graphene film and 3D graphene foam		*In vitro* murine BV2 microglial cell line	Inflammatory behavior of significantly reduced on graphene; significantly lower on 3D foam vs. 2D film	Song et al. (2014)
PLA nanofiber scaffolds with rat NGF in hyaluronate hydrosol engrafted with IL-4 plasmid-loaded aldehyde cationic liposomes	IL-4 plasmid-loaded aldehyde cationic liposomes	*In vivo* implanted in rats with acute spinal cord injury	Downregulated acute microglial inflammatory response and reduced glial scar formation	Xi et al. (2020)
PCL/PSA hybrid nanofiber scaffolds encapsulating MP	MP	*In vivo* implanted into rats with spinal cord transection	Inhibited microglial inflammatory activation; reduced secretion of TNF-ɑ and IL-6	Zhang et al. (2018)
Nanostructured self-healing hyaluronan and chitosan hydrogel scaffold		*In vivo* injected into rat brain striatum	Negligible microglial activation or neuroinflammation	Liu et al. (2020)
Oligodendrocytes				
PLLA nanofibers	Coated with PLL	*In vitro* rodent oligodendrocyte progenitors	Oligodendrocyte differentiation and ensheathment (myelination)	Lee et al. (2012)
Polystyrene nanofibers	Coated with PLL	*In vitro* rodent oligodendrocyte progenitors	Oligodendrocyte differentiation and ensheathment (myelination)	Lee et al. (2013)
FGLmx nanofibrous self assembling peptide scaffolds	FGL	*In vitro* rat oligodendrocyte progenitors	Oligodendrocyte differentiation	Wang et al. (2015)
PCL nanofiber platforms coated with laminin	Laminin coating	*In vitro* human pluripotent stem cell derived oligodendrocyte precursors	Cell orientation guided to resemble that of spinal cord *in vivo*	Hyysalo et al. (2017)
Hybrid PCL-gelatin nanofiber scaffold with polyaniline graphene	T3	*In vitro* rat bone marrow stem cell derived neural stem cells	Oligodendrocyte differentiation	Rasti Boroojeni et al. (2020)
PCL nanofibers loaded with PDGF-AA, FGF2, BMP2 and BMP4 and coated with laminin	PDGF-AA, FGF1, BMP2, BMP4; laminin coated	*In vitro* primary mouse oligodendrocytes	Myelination of nanofibers	Enz et al. (2019)
Polyethersulfone nanofiber meshes	Laminin coated	*In vitro* primary rat hippocampal derived neural stem cells	Oligodendrocyte differentiation	Christopherson et al. (2009)
PCL nanofibers coated with graphene oxide and laminin	Graphene oxide and laminin coating	*In vitro* primary rat neural stem cells	Oligodendrocyte differentiation	Shah et al. (2014)
PCL nanofibers loaded with miR-219, miR-338-3p and miR-338-5p, and coated with laminin	MicroRNA (miR-219, miR-338-3p and miR-338-5p); laminin coating	*In vitro* primary rat oligodendrocyte precursors	Differentiation and maturation into oligodendrocytes	Diao et al. (2015)
PCL nanofibers co-polymerized with 50% gelatin	Gelatin	*In vitro* neonatal rat oligodendrocyte precursor cells	Enhanced differentiation and myelination	Li et al. (2014)
PCL-PSA hybrid nanofiber scaffold	Methylprednisolone	*In vivo* rat transected spinal cord injury	Increased survival of oligodendrocytes and axonal myelination	Zhang et al. (2018)
PCL nanofibers coated with laminin	Laminin coating	*In vitro* human induced pluripotent cell derived oligodendrocytes	Induction of myelination	Ehrlich et al. (2017)
RAD16-I self-assembling peptide nanofiber scaffolds containing embryonic hippocampal neural progenitor cells	Embryonic hippocampal neural progenitor cells	*In vivo* adult rats with spinal cord dorsal column transection	Oligodendrocyte differentiation	Guo et al. (2007)
IKVAV self-assembling peptide nanofibrous scaffolds	IKVAV	*In vivo* female mice with dorsoventral compression induced spinal cord injury	Reduction in oligodendrocyte death during astrogliosis	Tysseling-Mattiace et al. (2008)
Fibrin nanofibrous scaffolds loaded with NT-3	NT-3	*In vitro* mouse embryonic stem cell derived neural progenitor cells	Oligodendrocyte differentiation	Willerth et al. (2008)
Collagen-PCLEEP hybrid nanofibrous scaffold loaded with NT-3	NT-3	*In vivo* hemi-cervical incision induced rat spinal cord injury	Extensive oligodendrocyte remyelination	Nguyen et al. (2017)

Nanocomposite hydrogel constructs are used frequently in neural tissue engineering to promote cell adhesion and proliferation, incorporate guidance cues, and provide electrical conductivity in the tissue-supporting scaffold (Madhusudanan et al., 2020). These properties make them especially attractive as injectable materials to deliver APIs to astrocytes. Conductive hydrogels with nanoparticles (Yang et al., 2022) or nanosheets (Chen et al., 2022) following stimulated spinal cord injury demonstrated decreases in GFAP-labeled astrocytes, as well as decreases in chondroitin sulfate proteoglycans and increased neuronal markers (Table 2). Studies using nanofiber hydrogels (NFH) have shown different outcomes, with NFH alone demonstrating no induction of astrocytes (Gonzalez et al., 2022), while increased amounts of astrocytes at the injury site were seen using an NFH construct combined with BMSCs (Li et al., 2020; Haggerty et al., 2022). Nanoparticle hydrogel composites have also shown varying results (Serafin et al., 2022). Hydrogel nanohybrids releasing NGF, diacerein, or chondroitinase ABC have reduced astrocyte activity (Raspa et al., 2021; Gao et al., 2022; Xing et al., 2023), demonstrating the potential of these API-releasing nanomaterials to improve astrocyte outcomes.

### 2.3. Nanoparticle API delivery to microglia

Microglia constitute 5%–10% of total brain cells and are the only true CNS parenchymal macrophages (Aguzzi et al., 2013). Upon CNS injury or disease, microglia adopt an “amoeboid” morphology and are responsible for phagocytosis and elimination of microbes, dead cells, and protein aggregates, and the secretion of soluble factors, including chemoattractants, cytokines, and neurotrophic factors (Colonna and Butovsky, 2017; Li and Barres, 2018). These polarized cells were traditionally categorized as having either toxic (M1) or protective (M2) states; however, accumulating evidence suggests microglial polarization is complex and multidimensional (Ransohoff, 2016a). In fact, single cell sequencing suggests that depending upon their anatomical compartment and pathological environment, microglia display an entire spectrum of functional states, ranging from highly inflammatory and phagocytic to anti-inflammatory and neuroprotective (Sankowski et al., 2022) Persistent pro-inflammatory microglial activation is a component of almost all neurodegenerative diseases (Ransohoff, 2016b). Because of this, many APIs have been developed to target microglia in order to improve outcomes in CNS disorders or after injury. This has prompted researchers to employ nanomaterials as an engineering approach to amplify further the impact of APIs designed for microglia.

NPs, including polymer, dendrimer, lipid, extracellular vesicles, and inorganic nanoparticles, have been designed to deliver APIs to microglia in many different CNS disorders and studied in various models, including autism spectrum disorder, cerebral palsy, neuropathic pain, SCI, Alzheimer’s, experimental autoimmune encephalomyelitis (EAE), TBI, retinal degeneration, Rett syndrome, and stroke (Iezzi et al., 2012; Sharma et al., 2017, 2020; Ganbold et al., 2020; Khoury et al., 2020; Liao et al., 2020; Gao et al., 2021; Kim et al., 2021; Liu et al., 2021; He et al., 2022; Sepasi et al., 2023) (Table 1). API payloads range from small molecule drugs to proteins, peptides, and siRNA (Lee et al., 2021; Liu et al., 2021; Hernando et al., 2022; Zhang M. et al., 2023). Most NP API therapies aim to modulate the microglial inflammatory response, polarizing microglia towards the more neuronally protective M2 phenotype to alleviate the inflammatory response and improve functional outcomes (Papa et al., 2013, 2016; Lu et al., 2014; Nance et al., 2015, 2017; Saxena et al., 2015; Kim et al., 2017; Wang Y. et al., 2018; Ellert-Miklaszewska et al., 2019; Cahalane et al., 2020; Cho et al., 2021; Xiao et al., 2021; Baghbanbashi et al., 2022; Ganbold et al., 2022; Guo et al., 2022; Hollinger et al., 2022; Shin et al., 2022; Ishida et al., 2023; Kalashnikova et al., 2023; Pu et al., 2023). NP API delivery to microglia has been shown to improve functional outcomes in many *in vivo* models of CNS disorders (Table 1), demonstrating that these cells have important implications across CNS pathologies and that modulating their response to injury and disease using NPs has immense potential in improving clinical outcomes.

### 2.4. Nanomaterial API delivery to microglia

Nanomaterial strategies targeting microglia are focused on nanofibrous scaffolds and hybrid nanostructured materials, often with an immunomodulatory payload to polarize microglia towards an anti-inflammatory phenotype in order to promote neuronal protection and repair (Table 2). Microglia have diverse, complex reactions to nanomaterials. 3D biodegradable hybrid inorganic nanoscaffolds modulated microglia *in vivo* to reduce scar formation during stem cell transplantation therapy for SCI (Yang et al., 2018). PCL nanofiber scaffolds coated with self-assembled colloidal graphene implanted in the striatum or subventricular zone of adult rats promoted reduced microglial infiltration (Watson et al., 2017). On the other hand, when primary microglia were cultured on poly(trimethylene carbonate-co-1-caprolactone) nanofibrous scaffolds, there was a reduction in phagocytic capacity, which indicates an inflammatory phenotype (Pires et al., 2015). Microglia were studied with engineered self-assembling (RADA)_4_-IKVAV peptide nanoscaffolds, and *in vitro* remained viable, phagocytosed the matrix, and remained ramified with high levels of TNF-ɑ and IL-1b and NO expression. When injected intracerebrally, however, the nanoscaffold did not lead to microglial migration, proliferation, or microglia-induced scarring (Koss et al., 2016). The inflammatory behavior of BV2 microglia was significantly reduced when interfaced with graphene nanomaterials compared to conventional polystyrene tissue culture substrates, and 3D graphene foams elicited a significantly milder neuroinflammatory response compared to a 2D graphene film (Fabbri et al., 2021). Nanostructured self-healing hyaluronan and chitosan hydrogel scaffolds injected into the rat brain striatum had negligible microglial activation or neuroinflammation (Liu et al., 2020).

The ability of nanomaterials to alter microglial response led researchers to include APIs during development to add a further level of control. Glial cell-derived neurotrophic factor (GNDF)-loaded polydopamine (PDA) nanoparticle-based anisotropic gelatin scaffolds efficiently deliver PDA nanoparticles to scavenge reactive oxygen species and promote the M2 anti-inflammatory polarization in the murine BV2 microglial cell line (Ma et al., 2023). Poly(lactic acid) nanofiber scaffolds with incorporated rat NGF in hyaluronate hydrosol were engrafted with immunoregulatory IL-4 plasmid-loaded aldehyde cationic liposomes and implanted into rats with acute SCI, resulting in a downregulated acute microglial inflammatory response and reduced glial scar formation (Xi et al., 2020). PCL/PSA hybrid nanofiber scaffolds encapsulating methylprednisolone (MP) implanted after transection SCI inhibited microglial inflammatory activation as evidenced by reduced secretion of TNF-ɑ and IL-6 (Zhang et al., 2018). As more advanced nanomaterials are developed, the ability to deliver APIs that modulate the microglial response has therapeutic potential in many CNS disorders.

### 2.5. Nanoparticle API delivery to oligodendrocytes

Oligodendrocytes generate myelin to increase the speed of propagation of axon potentials and provide metabolic support to neurons in the CNS (Simons and Nave, 2015). Unfortunately, oligodendrocytes are vulnerable to reactive oxygen species, hydrogen peroxide, and excitotoxicity from glutamate, and as such, are detrimentally impacted in a range of CNS disorders (Matute et al., 1997; Juurlink et al., 1998; Kuhn et al., 2019; Kenigsbuch et al., 2022; Pandey et al., 2022). The most common causes of oligodendrocyte death in the CNS are trauma, ischemia, or autoimmune attacks, such as multiple sclerosis. However, white matter pathology is also characteristic of other CNS diseases, including Alzheimer’s (Love, 2006; Fancy et al., 2011; Assinck et al., 2017; McAleese et al., 2017). Remyelination is a natural regenerative process that has been shown to prevent neurodegeneration and restore function (Duncan et al., 2009, 2018). Therefore, APIs have been studied in order to promote oligodendrocyte remyelination in CNS trauma and disease. Nanomaterial design for API delivery to oligodendrocytes is being studied to capitalize on the synergy between the advantageous properties of the API and those of the material (Russell and Lampe, 2017; Murphy and Lampe, 2018).

Nanoparticles, including polymer, lipid, extracellular vesicles, and inorganic nanoparticles, have been designed to deliver APIs to oligodendrocytes in models of TBI, EAE, focal CNS demyelination, cuprizone-induced demyelination, SCI, and ischemia (Table 1) (Rittchen et al., 2015; Kumar et al., 2018; Lin et al., 2019; Zhang et al., 2019; Osorio-Querejeta et al., 2020; Robinson et al., 2020; Pu et al., 2021). API payloads range from small molecule drugs to proteins, peptides, and miRNA (Fressinaud et al., 2020; Farhangi et al., 2023; Moura et al., 2023). The goal of most NP API therapies directed towards oligodendrocytes is to reduce myelin loss and induce remyelination after injury or disease in order to improve functional outcomes (Table 1). Importantly, NP API delivery to oligodendrocytes has been shown to rejuvenate myelin and improve outcomes in *in vivo* models of CNS injury and demyelinating disorders (Table 1). While NP API delivery to oligodendrocytes is the least studied of the three most prominent glia in the CNS, the functional benefits demonstrate the potential for developing these NP therapies to improve myelin outcomes in many different CNS pathologies in order to push these treatments towards the clinic.

### 2.6. Nanomaterial API delivery to oligodendrocytes

Nanomaterial strategies that target oligodendrocytes have focused on engineered nanofibrous materials due to their ability to provide an axon-like substrate to promote oligodendrocyte differentiation and myelination. Two fundamental studies pioneered this approach by demonstrating that rat oligodendrocyte progenitor cells (OPCs) cultured on electrospun nanofibers of diameter 500-800 nm proliferated and differentiated into oligodendrocytes and ensheathed the fibers, resembling myelination (Lee et al., 2012). The same group also reported similarly compacted myelination on polystyrene electrospun nanofibers cultured with rodent oligodendrocytes (Lee et al., 2013). Further studies have shown that nanofibers can guide oligodendrocyte orientation that more closely resembles *in vivo* morphologies, preferentially drive neural stem cells to oligodendrocytes, induce compact myelination, and protect oligodendrocytes following traumatic CNS injury (Tysseling-Mattiace et al., 2008; Cao et al., 2009; Li et al., 2014; Shah et al., 2014; Wang et al., 2015; Ehrlich et al., 2017; Hyysalo et al., 2017; Tupone et al., 2021; Zhang et al., 2022) (Table 2). The ability to mimic the *in vivo* environment and alter oligodendrocyte response has led to the design of nanomaterials that release APIs to act more specifically on these glia.

Further, hybrid PCL-gelatin nanofiber scaffolds, combined with polyaniline graphene nanocomposites, were incorporated in gelatin to lend conductive properties similar to axons. Chitosan nanoparticles loaded with T3 were incorporated into PCL for sustained release, and these nanohybrids led to the differentiation of rat bone marrow-derived neural stem cells towards an oligodendrocyte lineage with high expression of PDGFRα, O4. Olig2, O1, MOG, and MBP (Rasti Boroojeni et al., 2020). Primary oligodendrocytes isolated from B16 mice were able to myelinate aligned PCL nanofibers that released PDGF-AA, FGF2, BMP2, and BMP4 (Enz et al., 2019). PCL nanofibers loaded with miR-219, miR-338-3p, and miR-338-5p enhanced the differentiation of primary rat oligodendrocyte progenitor cells and their maturation into RIP+ oligodendrocytes (Diao et al., 2015).

Moreover, when hybrid PCL-PSA (polysialic acid) nanofiber scaffolds encapsulating glucocorticoid methylprednisolone were implanted into a transected rat SCI, the methylprednisolone delivered by the hybrid scaffold led to increased survival of oligodendrocytes and enhanced axonal myelination (Zhang et al., 2018). NT-3 is another API that has been used to act on oligodendrocytes, and fibrin nanofibrous scaffolds releasing NT-3 increase oligodendrocyte differentiation of neural progenitor cells (Willerth et al., 2008), while PCLEEP(PCL-co-ethyl ethylene phosphate)-collagen hybrid nanofibrous scaffolds releasing NT-3 showed extensive oligodendrocyte remyelination with MAG+ structures when implanted into a hemi-cervical incision induced rat spinal cord injury (Nguyen et al., 2017). Future oligodendrocyte-targeting nanomaterial design will seek to devise API-releasing strategies that specifically improve re-myelination after injury and improve myelin integrity in nervous system disorders.

## 3. Discussion

Nanomaterials designed to deliver APIs to CNS glia are beginning to emerge as viable therapies to improve outcomes in CNS disorders or after CNS injury. While many API-releasing nanomaterials are still being designed to focus their action on neurons (Kwon et al., 2016; Bruggeman et al., 2018; Zuidema et al., 2020), there is growing evidence that glia should not be overlooked as targets to improve outcomes in CNS injury and disorders (Tables 1, 2). However, in order for nanomaterial-mediated API delivery to glia to become a standard clinical intervention, further advances in engineering such materials are necessary.

Nanoparticle-mediated API delivery holds promise as a systemically administered approach to treat neurological disorders and CNS injuries where direct implantation into the site of action would be detrimental. For such treatments to become commonplace, one of the main areas of improvement is in traversing the BBB and delivering APIs directly to the relevant site of action. This will require a greater understanding of the mechanisms of nanoparticle permeation into the brain, including the importance of NP composition, size, charge, and shape, engineering the adsorbed biomolecular corona to not obstruct NP targeting, targeting the proper cell type once the NPs enter the brain, design of better-targeting moieties on the external surface of nanoparticles through such processes as *in vivo* phage display screening, exact API release timelines that induce desired outcomes, and, importantly, a more complete understanding of how to modulate glia to produce desired clinical outcomes (Salvati et al., 2013; Mann et al., 2016; Furtado et al., 2018; Waggoner et al., 2023; Wu et al., 2023). More personalized therapies can be envisioned, where each individual may respond to NPs differently. This may require a battery of different NP constructs to first be administered systematically, and once it is known which NP accumulates to the desired location, potential by using an imaging modality such as magnetic resonance imaging, then that NP construct can be incorporated with the desired API and delivered to the individual. Still, much research is needed to make NP-delivered APIs that act on glia a standard therapy to treat CNS disorders and injuries.

Nanomaterial-mediated API delivery has shown promise in areas where surgical intervention or injection into the site of action to act on glia can be used. These combinatorial nanomaterial-based therapies can simultaneously provide biophysical and biochemical cues to glial cells, eliciting their bioactive responses to facilitate robust neuronal repair and protection in the CNS. For these therapies to be used in the clinic, advances in API release paradigms must be realized, nanomaterial modulation of glia needs to be better understood, surgical implantation techniques optimized, degradation of the implanted material engineered based on the application, and the immune response accounted for not to impart adverse clinical outcomes (Nunes et al., 2012; Huang et al., 2017; Dai et al., 2021). We also envision the potential for nanomaterial therapies to be tailored to each patient to maximize therapeutic efficacy and minimize off-target adverse effects – by varying API release rates, compositions and coatings in nanomaterials design, and even the timeline of the surgical intervention. As advances in NPs, nanomaterials, and API design for targeting glia continue to be realized, there are many avenues for such therapies to improve clinical outcomes in CNS disorders and after CNS injury.

## Author contributions

JS: Conceptualization, Investigation, Writing – original draft, Writing – review & editing. AH: Conceptualization, Funding acquisition, Investigation, Writing – original draft, Writing – review & editing. RG: Conceptualization, Funding acquisition, Project administration, Supervision, Writing – review & editing. JZ: Conceptualization, Funding acquisition, Project administration, Supervision, Writing – original draft, Writing – review & editing.
